# Fibroblast activation protein is a cellular marker of fibrotic activity in canine idiopathic pulmonary fibrosis

**DOI:** 10.3389/fvets.2024.1416124

**Published:** 2024-07-31

**Authors:** Elodie Rizzoli, Constance de Meeûs d'Argenteuil, Aline Fastrès, Elodie Roels, Pierre Janssen, Ellen Puré, Mutien-Marie Garigliany, Thomas Marichal, Cécile Clercx

**Affiliations:** ^1^Department of Companion Animal Clinical Sciences, Fundamental and Applied Research for Animals and Health (FARAH), Faculty of Veterinary Medicine, University of Liège, Liège, Belgium; ^2^Department of Morphology and Pathology, FARAH, Faculty of Veterinary Medicine, University of Liège, Liège, Belgium; ^3^Laboratory of Immunophysiology, GIGA Institute, University of Liège, Liège, Belgium; ^4^Department of Functional Sciences, FARAH, Faculty of Veterinary Medicine, University of Liège, Liège, Belgium; ^5^Department of Biomedical Sciences, School of Veterinary Medicine, University of Pennsylvania, Philadelphia, PA, United States; ^6^Walloon Excellence in Life Sciences and Biotechnology (WELBIO) Department, WEL Research Institute, Wavre, Belgium

**Keywords:** dog, lung, fibrosis, idiopathic pulmonary fibrosis, fibroblast activation protein, immunohistochemistry, West Highland white terrier

## Abstract

Canine idiopathic pulmonary fibrosis (CIPF) is a progressive fibrotic interstitial lung disease of unknown etiology, afflicting aging West Highland white terriers (WHWTs) and leading to progressive respiratory failure. Fibroblast activation protein (FAP), a protease overexpressed in many cancers, is upregulated in idiopathic pulmonary fibrosis in humans. The aim of this study was to investigate FAP as a marker of active fibrosis in lung biopsies from WHWTs affected with CIPF, as well as the potential of plasmatic FAP as a biomarker. After establishing a scoring system to evaluate the severity and activity of fibrosis on histopathological lung sections, anti-FAP immunohistochemistry was performed on healthy and CIPF samples. FAP expression was characterized using both visual and digital quantitative pathology software analyses and then correlated to fibrosis severity and activity. Levels of plasmatic FAP in WHWTs affected with CIPF were measured by enzyme-linked immunosorbent assay and compared with healthy dogs. Lung samples from 22 WHWTs affected with CIPF were collected. According to the fibrosis scoring system, they were classified as cases of mild (5), moderate (9) and severe (8) fibrosis and were attributed scores of fibrosis activity. Fifteen healthy lung samples were classified as non-fibrotic. Healthy lung samples were FAP-negative, whereas fibroblasts were FAP-positive in 20 CIPF samples. FAP immunohistochemical expression correlated mildly with fibrosis severity (*p* < 0.05; *R*^2^ = 0.22) but highly with fibrosis activity scores (*p* < 0.001; *R*^2^ = 0.68). Digital image analysis detected a higher percentage of FAP-positive cells in areas of active fibrosis (*p* < 0.001) and FAP-positive cells were distributed outside mature fibrosis lesions, clustered in active fibrosis areas or scattered within alveolar septa. On the other hand, plasmatic FAP was significantly lower in dogs affected with CIPF compared with healthy dogs (*p* < 0.01). In conclusion, this study provides a valuable histological scoring system to assess the severity and activity of fibrosis in CIPF. It demonstrates that FAP is a good cellular marker of fibrotic activity in CIPF, and thus constitutes a promising target to be exploited for diagnostic and therapeutic applications. Additionally, it suggests that plasmatic FAP, although non-specific, could be altered in CIPF.

## 1 Introduction

Canine idiopathic pulmonary fibrosis (CIPF) is a progressive fibrotic interstitial lung disease of unknown etiology, affecting the West Highland white terrier (WHWT) breed leading to progressive respiratory insufficiency, mimicking idiopathic pulmonary fibrosis (IPF) in humans (1, 2). Currently, there are neither consistent diagnostic or prognostic biomarker nor curative treatment options available for this disease (1, 2).

Fibroblast Activation Protein (FAP), also known as seprase, is a cell surface protease which exhibits both dipeptidyl peptidase activity and endopeptidase activity (3). Among substrates of the endopeptidase activity, FAP cleaves denatured type 1 collagen, thus participating in extracellular matrix (ECM) remodeling (3). The protease also exists as a soluble circulating form called antiplasmin-cleaving enzyme (APCE) (4). FAP is specifically expressed in areas of physiological and pathological active tissue remodeling, including wound healing and scar formation in mammals (5). FAP is usually undetectable in normal tissue (5), although low basal levels have been measured in human adipose tissue, liver and plasma (6, 7).

In human IPF, immunohistochemical studies on lung biopsies showed that FAP is strongly expressed in areas of lung fibrosis, namely in fibroblast foci and interstitium, and is positively correlated with the severity of fibrosis (8, 9). In humans, FAP is also upregulated in other fibrotic diseases (10–12) as well as non-fibrotic diseases (13, 14), and, importantly, in various types of cancers. Indeed, it is expressed in over 90% of carcinomas, including among others non-small-cell lung carcinoma (15–17), colorectal (18, 19), esophageal (20), breast (21–23), and renal (24) cancer. The protease is mainly present in cancer associated fibroblasts, but can also be expressed in other cells in the tumor microenvironment [immune (25) cells or endothelial (19) cells] or in epithelial tumor cells (17, 19). In dogs, overexpression of FAP has already been demonstrated in the stroma of mast cell tumors and mammary carcinomas (26, 27) as well as in the right atrium of beagle dogs with induced atrial fibrillation (28). Moreover, overexpression of the FAP gene has been observed in post-mortem lung biopsies from WHWTs affected with CIPF compared with healthy controls based on microarray analysis and quantitative reverse transcriptase polymerase chain reaction (29).

Recently, FAP-targeted positron emission tomography (PET) imaging using a FAP inhibitor (FAPI) has been described as a non-invasive sensitive tool for advanced tumor staging and monitoring and has a promising potential owing to its ability to accurately depict most malignant tumors (30). Beyond its application in neoplastic disorders, there have been encouraging reports suggesting the utility of FAPI PET in non-neoplastic conditions such as respiratory or cardiac diseases including IPF (9, 31–33). Indeed, the uptake of FAP-targeted tracers (labeled with either ^68^Ga or ^18^F) in IPF patients is higher than in healthy volunteers, and also seems to be positively correlated to the pulmonary function decline (9, 33).

Given the potential role of FAP in the pathogenesis of fibrosis and cancer, several therapeutic strategies seek to target this protein, from selective inhibitors (34) to anti-FAP chimeric antigen receptor (CAR)-T cells (35) or even recent theragnostic ligands (36). However, none of these FAP-based therapeutic approaches have been approved in humans yet.

If FAP appears to be a specific marker of active fibrosis in dogs with CIPF, it could represent both an interesting diagnostic and monitoring marker of the disease and importantly, a potential therapeutic target. Therefore, the aim of this study was to gain insight into the implication of FAP in the pathophysiology of CIPF and to confirm its potential as a marker of disease activity. We hypothesized that FAP is expressed in lungs of WHWTs affected with CIPF, as well as in the stroma of canine lung cancers, used as positive controls, but not in healthy lungs. Anti-FAP immunohistochemistry (IHC) staining was thus performed on sections of lung biopsies from WHWTs affected with CIPF, dogs with lung cancer and dogs without pulmonary disease. The pattern of FAP expression was characterized according to the pattern of severity and activity of fibrosis, using both visual and digital quantitative pathology software analyses. Finally, the potential of circulating FAP as a biomarker of CIPF was investigated by measuring the levels of plasmatic FAP in WHWTs affected with CIPF in comparison with healthy dogs.

## 2 Materials and methods

### 2.1 Lung sample collection

For this cross-sectional observational study, lung biopsies were obtained from 22 WHWTs affected with CIPF (median age of 12.4 years; range 10.3–15.6; 10 females and 12 males), 15 dogs of various breeds [WHWT (4), Beagle (3), Yorkshire Terrier (3), mixed breed (2), American Staffordshire Terrier, Bull Terrier, Leonberger, and Shih Tzu] exempt from lung disease (median age of 13.2 years; range 7.3–16.8; 4 females and 11 males) and 7 dogs of different breeds [WHWT (4), mixed breed (2), Weimaraner] with lung neoplasia (median age of 12.0 years; range 8.2–14.2; 5 females and 2 males). In WHWTs, CIPF diagnosis was based on clinical signs, physical examination, 6-min walk test, hematology, serum biochemistry, arterial blood gas analysis, cardiac ultrasonography, thoracic high-resolution computed tomography, bronchoscopy and analysis of bronchoalveolar lavage fluid (1, 37). CIPF and healthy WHWTs were recruited as part of a longitudinal study conducted at the University of Liège and approved by the Animal Ethics Committee of the University of Liège (approval #20–2245). Healthy controls were euthanized for reasons unrelated to the study and had no respiratory clinical signs and normal lung histopathology. Five post-mortem lung biopsies were collected: one in the periphery of the right cranial and accessory lobes, two in the periphery of the right diaphragmatic lobe – one ventrally and one dorsally – and one centrally in the right middle lobe. Biopsies of pulmonary neoplasia, collected after either lobectomy or necropsy, were also retrieved. All biopsies were fixed in formalin 10% and embedded in paraffin until further use. All samples were obtained with informed owner consent.

### 2.2 Histopathology and fibrosis scoring

Formalin-fixed, paraffin-embedded specimens were sliced into 5 μm sections with a motorized microtome (Microm HM355S, Thermo Fisher Scientific). Hematoxylin and eosin (HE) staining was initially performed. All slides were evaluated by the first author and by a diplomate of the European College of Veterinary Pathology (MMG), who were blinded to the clinical records. For each healthy and CIPF case, one representative section was selected and additional serial slides were stained with Masson’s trichrome and Picro Sirius red for further use. All sections were digitalized with NDP NanoZoomer (Hamamatsu) and Picro Sirius red slides were additionally digitalized under polarized light with ZEISS Axioscan 7.

For each selected section, a scoring system of fibrosis was applied, based on both HE and Masson’s trichrome. For this purpose, a list of criteria was established, as detailed in Table 1, based on previously reported histopathological examinations of CIPF lung sections (37, 38) and the latest consensus for histopathological diagnosis of human IPF (39). Categories of criteria included the pattern of interstitial fibrosis (evaluating the severity of fibrosis in the subpleural area, in peribronchiolar area as well as within alveolar septa) based on Masson’s trichome stained sections, the maturity of fibrosis and its extent over the section (based on HE and Masson’s trichrome), and alveolar epithelial and luminal changes (based on HE). Regarding the maturity of fibrosis, each case was assigned a score of active fibrosis from 0 to 3 reflecting the proportion of the section affected by active fibrosis, which was defined as immature, highly cellular, fibroblast-dominant fibrosis (38). Another score from 0 to 3 was attributed according to the contribution of mature fibrosis, which was defined as inactive fibrosis and characterized by dense collagen deposition and low cellularity consisting of a few fibrocytes (38). An overall grade of fibrosis severity was attributed according to the total score as follows: 0–3 (non-fibrotic), 4–7 (mild), 8–11 (moderate) and 12–16 (severe). For lung tumor cases, the histopathological diagnosis was established as precisely as possible based on medical records and HE stained slides.

**Table 1:** Scoring system used to evaluate fibrosis in canine idiopathic pulmonary fibrosis lung biopsies.

Histopathological features	Criteria	Scores			
		0	1	2	3
Interstitial fibrosis pattern					
Subpleural	Increase in pleural width	0	2x	≥3x	NA
Peribronchiolar fibrous metaplasia	Smooth muscle over lamina propria and adventitia thickness ratio	>0.34	≤0.34	NA	NA
Diffuse	Increase in septa width by fibrosis	0	2x	3–4x	≥5x
	Atelectasis, alveolar distortion, consolidation, and/or honeycombing	Absent	NA	NA	Present
Maturity of fibrosis					
Immature, active, cellular, fibroblast-dominant*	Proportion of the section affected by active fibrosis	0%	1–33%	34–66%	≥67%
Mature, inactive, fibrous, few fibrocytes*	Proportion of the section affected by mature fibrosis	0%	1–33%	34–66%	≥67%
Alveolar epithelial and luminal changes					
Type II pneumocyte hyperplasia/bronchiolar metaplasia	Alveolar epithelium	Normal	Type II pneumocyte hyperplasia and/or atypia	Pseudo-stratification	NA
Numerous alveolar macrophages	Alveolar macrophages count per alveolar space	1–2	≥3	NA	NA

The grade of severity of fibrosis was attributed by calculating the sum of the score attributed for each criteria: 0–3 (non-fibrotic), 4–7 (mild), 8–11 (moderate) and 12–16 (severe). *As the sum of both percentages cannot exceed 100% (whole section), the sum of the scores from these 2 categories is maximum 4.

### 2.3 Tissular FAP immunohistochemistry

#### 2.3.1 Staining

Anti-FAP IHC was performed on additional serial sections of formalin-fixed, paraffin-embedded biopsies of CIPF, healthy lungs and lung cancers, which were used as positive controls. The slides were deparaffinized in xylene and rehydrated in graded alcohol series. Antigen retrieval was performed using 10 mM sodium citrate buffer for 5 min at 100°C. Slides were washed at room temperature and hydrated in Phosphate-Buffered Saline. Endogenous peroxidase activity was blocked with 3% hydrogen peroxide incubation for 30 min. Sections were then washed with distilled water. Nonspecific antibody binding was blocked by incubation for 30 min in a blocking buffer containing 0.5% blocking reagent provided in the TSA Plus DNP kit (Akoya Biosciences #NEL747A001KT). Sections were incubated overnight at 4°C temperature with rabbit anti-human fibroblast activation protein alpha monoclonal primary antibody (1: 100, Abcam #ab207178, RRID:AB_2864720) or with rabbit isotype IgG control antibody (1:1600, Jackson ImmunoResearch Labs #011–000-003, RRID:AB_2337118) to later screen for non-specific staining. Biotinylated goat anti-rabbit secondary antibody (1,1000, Thermo Fisher Scientific #65–6,140, RRID:AB_2533969) was then incubated for 1 h at room temperature. The slides were incubated for 30 min with streptavidin-horseradish peroxidase (Invitrogen #S911), and signal was amplified using a TSA Plus DNP kit (Akoya Biosciences #NEL747A001KT). Signal development was achieved with a metal enhanced diaminobenzidine substrate kit (Thermo Fisher Scientific #34065). Slides were counterstained with hematoxylin for 30 s, then dehydrated and mounted. Each slide was digitalized using NDP NanoZoomer (Hamamatsu).

#### 2.3.2 Visual assessment of FAP expression

Two independent observers, including the first author and a diplomate of the European College of Veterinary Pathology (MMG), blinded to the histopathological diagnosis, assessed all healthy and CIPF digitalized sections to determine a staining index for the whole section, that represents the expression of FAP. There was 91% agreement between the two observers and the final index was obtained after a consensus was reached. An area of parenchymal lung was identified as FAP-positive if at least 25% of the cells exhibited FAP staining. The FAP expression index (from 0 to 3) was then attributed according to the percentage of the whole section occupied by FAP-positive areas. An index of 0 (no expression) was attributed if less than 1% of the section was occupied by FAP-positive areas, 1 (low expression) if FAP-positive areas occupied from 1 to 10% of the whole section, 2 (intermediate expression) from 11 to 50% and 3 (high expression) for more than 50%. In all CIPF cases, correlation analyses were conducted between the FAP expression index and the fibrosis severity score, as well as with the fibrosis activity score attributed during the scoring of fibrosis.

#### 2.3.3 Digital analysis of FAP expression

Whole slide images were analyzed with an open-source automated software analysis program for digital pathology (QuPath version 0.4.3) (40). Briefly, lesional areas were determined manually on the HE slides and classified into ‘active fibrosis’ or ‘mature fibrosis.’ Ten areas of 200,000 μm^2^ each representative of active fibrosis or mature fibrosis were selected. Automated tissue detection was performed in the lesional area to correct for alveolar blank spaces. Thereafter, for fibrosis quantification, built-in algorithms for pixel classification of QuPath and machine learning were used on sequential Picro Sirius red slides for measuring collagen content in lesional areas. The accuracy of collagen detection was then verified by assessing the same area digitalized under polarized light. On FAP-stained sections, the percentage of FAP-positive cells within the lung interstitium for the 20 areas was calculated by applying the deep learning algorithm StarDist method for cell nuclei segmentation and applying a single threshold to the cell detection to obtain positive cell detection. To visualize the spatial distribution of FAP positive cells in fibrotic areas, image superposition of Picro Sirius red slides and FAP-stained slides was done by using the Warpy extension in QuPath.

### 2.4 Plasmatic FAP measurement

#### 2.4.1 Test samples

For the plasmatic FAP measurement, we used plasma samples from the day of death of 6 WHWTs affected with CIPF for which positive FAP expression in the lungs was confirmed by the methods described above. They had a median age of 12.6 years (range 10.3–15.6; 3 females and 3 males). For the control group, we used the plasma leftover from the analysis of blood donations from 9 healthy canine blood donors of various breeds [Border Collie (4), Golden Retriever (3), Akita Inu, Bull Terrier] with a median age of 6.6 years (range 3.9–7.3), including 4 females and 5 males. Dogs were considered healthy based on the absence of clinical signs or physical exam abnormalities, a complete blood analysis and a screening for infectious diseases. In all dogs, blood was collected in a citrated tube before being centrifuged and plasma was isolated and stored at −80°C until the day of the experiment. The assay was performed in citrate plasma in all cases because it was the type of plasma that was available for the higher number of cases in our biobank. Plasma samples underwent maximum 2 freeze–thaw cycles before analysis. Plasma samples were diluted in 1% bovine serum albumin (BSA, Sigma #A7906) in Dulbecco’s Phosphate-Buffered Saline (DPBS) in four dilutions (1:50, 1:100, 1:200, 1:400) for titration. The reactivity of the assay with canine FAP was verified by using a homogenate of a FAP-rich metastasis of mammary carcinoma as positive control. A snap frozen biopsy of a lung metastasis of a mammary carcinoma that highly overexpressed FAP in IHC was homogenized using a previously described protocol (41). This canine FAP-containing solution was then diluted 1:5, 1:10, 1:25, 1:50 in 1% BSA DPBS in the assay for titration. Recombinant human FAP (Abcam #ab79623) with known concentration was used as positive control and standard. Negative control was 1% BSA DPBS.

#### 2.4.2 Assay

Plasma levels of FAP were measured using a double-antibody sandwich enzyme-linked immunosorbent assay (ELISA). First, 96-well microplates were coated with a mouse IgG monoclonal anti-canine FAP antibody (5.125 μg/mL, Puré lab, University of Pennsylvania, 4G5) that cross-reacts with human FAP (35) and incubated overnight at 4°C. The following day, plates were blocked with 1% BSA in DPBS for 1 h before test samples were added in duplicates and incubated overnight at 4°C with agitation. For detection, a biotinylated polyclonal sheep anti-human FAP antibody (0.4 μg/μl, R&D Systems #BAF3715, RRID:AB_2057508) was added and incubated for 90 min. Plates were then incubated with avidin horseradish peroxidase (1:1000 dilution, Thermo Fisher Scientific #18–4100-94) for 30 min, after which 3,3′,5,5′-tetramethylbenzidine (TMB, Life Technologies #SB02) was added. After a 10 min-incubation in the dark, reaction was stopped using 50 μL/well of H2SO4 1 M. Plates were read by an optical density reader (Multiskan FC, Thermo Fisher Scientific #51119000) set at 450 nm. Between each step until the chromogenic reaction, 3 to 5 rinses were performed with a wash solution of Tween-20 5% (Thermo Fisher Scientific #233360010) in DPBS.

Because of the lack of commercially available purified canine FAP protein to act as standard, the exact amount of soluble FAP in biological samples could not be calculated. Instead, we expressed results in endpoint titers (EPT). Using a plot of the optical density on the log base 2 of the dilutions, we defined the cutoff line at half the optical density of recombinant human FAP at 78.13 ng/mL concentration. The log2 of the endpoint titer was obtained from the point where the linear line crosses the cutoff line.

### 2.5 Statistical analysis

Statistical analyses were conducted using the R Commander interface (42) to the R statistical software. Normal distribution was assessed using Shapiro–Wilk normality test. For normally distributed data, parametric tests were used and results were expressed in mean and standard deviation. A Fisher test was used to verify homoscedasticity between groups. When variances were significantly different between groups, comparisons of means were performed using a Welsh two sample *t*-test. For non-normally distributed data, non-parametric tests were employed and results were expressed in median and interquartile range (P25-P75). For correlation analyses of non-normally distributed data, Spearman’s rank correlation rho test was used. For the comparison of medians between two groups, a Mann–Whitney test was used. Significance was established at a *p*-value lower than 0.05.

## 3 Results

### 3.1 Histopathological analysis

After scoring fibrosis in CIPF sections, five were characterized as mild (scores ranging from to 4 to 7), nine as moderate (scores ranging from 8 to 11) and eight as severe fibrosis (scores ranging from 12 to 16). Control lung sections were attributed scores from 0 to 3 and were considered as non-fibrotic. Seven cases of lung neoplasia, including six primary pulmonary adenocarcinomas and one metastasis of mammary carcinoma, served as positive controls for IHC.

### 3.2 Tissular FAP expression

**Figure 1:**
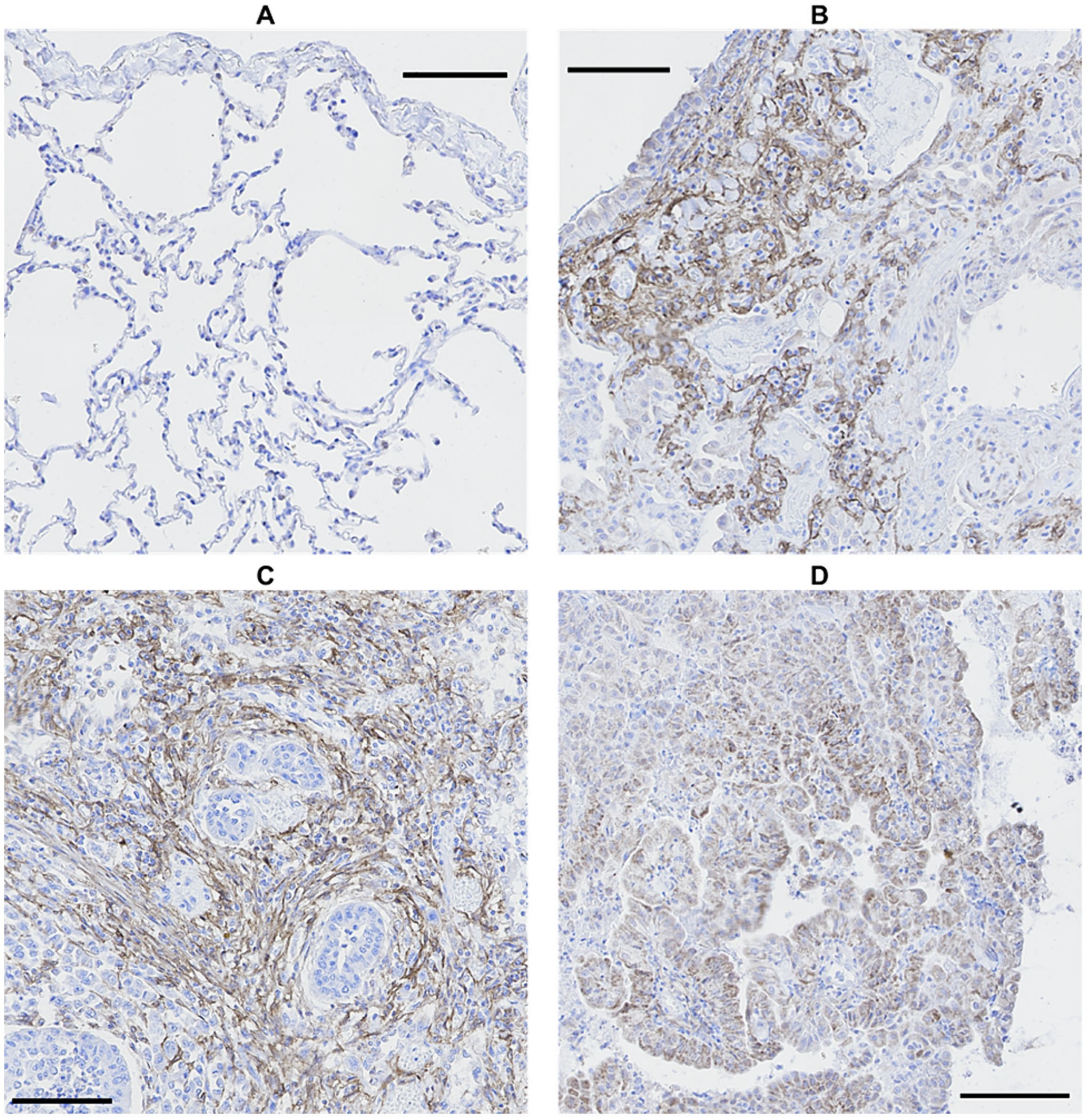
Comparison of FAP immunostaining in canine lung biopsies. No FAP expression in healthy lung **(A)** and high FAP expression in CIPF **(B)** and primary pulmonary adenocarcinoma, either in cancer-associated fibroblasts **(C)** or in cancer cells **(D)**. Immunoperoxidase-diaminobenzidine, hematoxylin counterstain (bar: 100 μm). FAP, fibroblast activation protein; CIPF, canine idiopathic pulmonary fibrosis.

**Figure 2:**
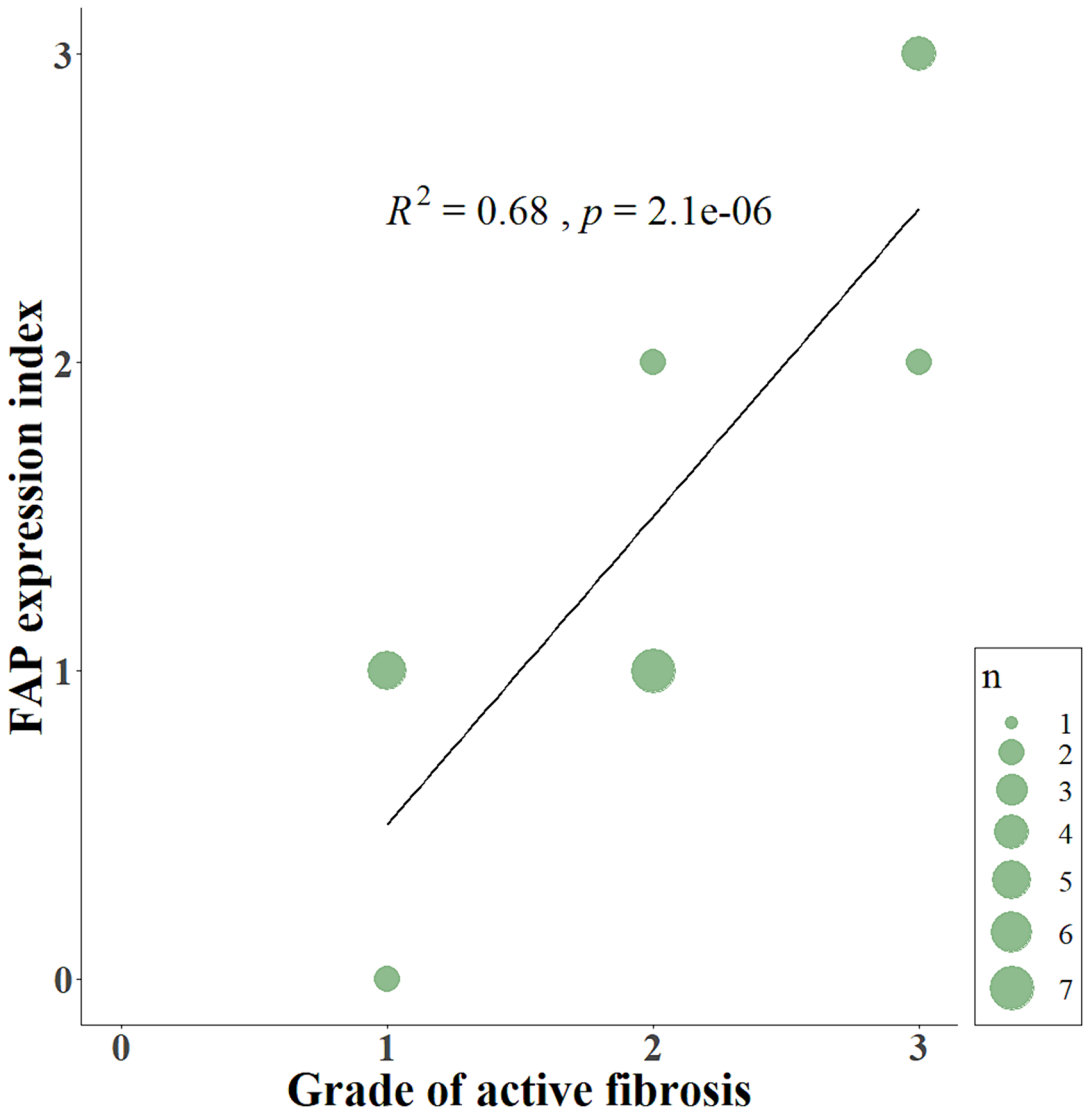
Scatterplot displaying the relationship between the FAP expression index and the grade of active fibrosis. The index of FAP expression (from 0 to 3) is positively correlated (*p* < 0.001; *R*^2^ = 0.68) to the score of active fibrosis (from 0 to 3) in lung sections of CIPF. FAP, fibroblast activation protein; CIPF, canine idiopathic pulmonary fibrosis; n, number of cases.

FAP was expressed in cells interpreted as fibroblasts in the lungs of 20 out of 22 WHWTs affected with CIPF. Using a visual semi-quantitative scoring system, an index of high, intermediate, low and zero FAP expression were attributed in, respectively, 4, 4, 12, and 2 WHWTs. Healthy lung biopsies from WHWTs and other breeds all had an index of zero FAP expression. In primary pulmonary adenocarcinomas and in the metastasis of mammary carcinoma, cancer-associated fibroblasts were strongly FAP-positive. Cancer cells were also FAP-positive in four out of six cases of primary adenocarcinoma. Figure 1 illustrates examples of FAP staining in lung sections. Within CIPF cases, there was a statistically significant but poorly relevant positive correlation (*p* < 0.05; *R*^2^ = 0.22) between the FAP expression index and the score of fibrosis severity. However, the index of FAP expression was highly positively correlated to the score of active fibrosis (*p* < 0.001; *R*^2^ = 0.68), as illustrated in Figure 2.

**Figure 3:**
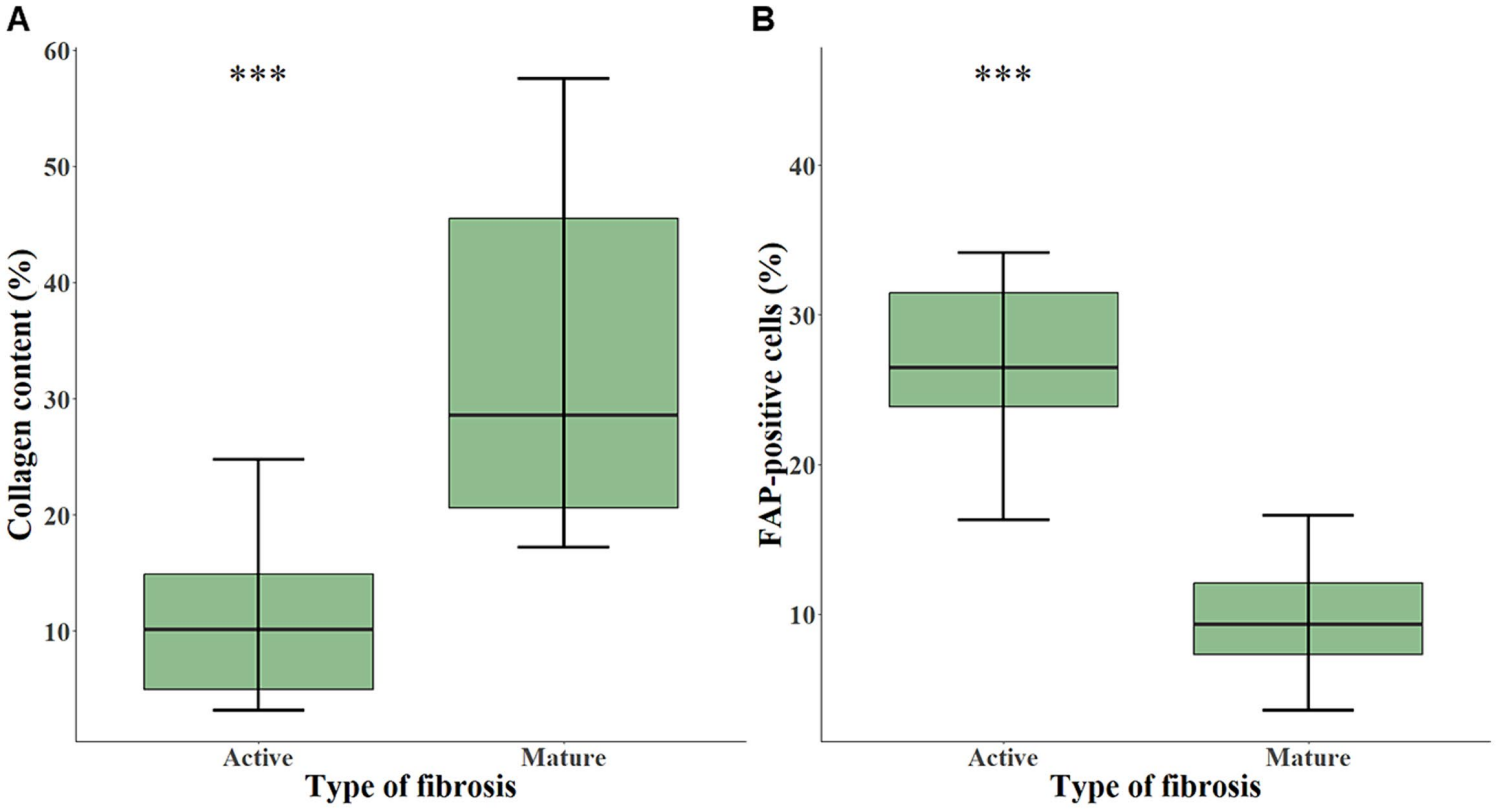
Box-and-whisker plots of collagen content **(A)** and of FAP-positive cells **(B)** in areas representing either active (*n* = 10) or mature fibrosis (*n* = 10), calculated with quantitative digital analysis in CIPF lung sections. The box represents the median and interquartile range. The whiskers represent the values within 1.5 times the interquartile range. Significance level: *** indicates a *p*-value below 0.001. FAP, fibroblast activation protein.

**Figure 4:**
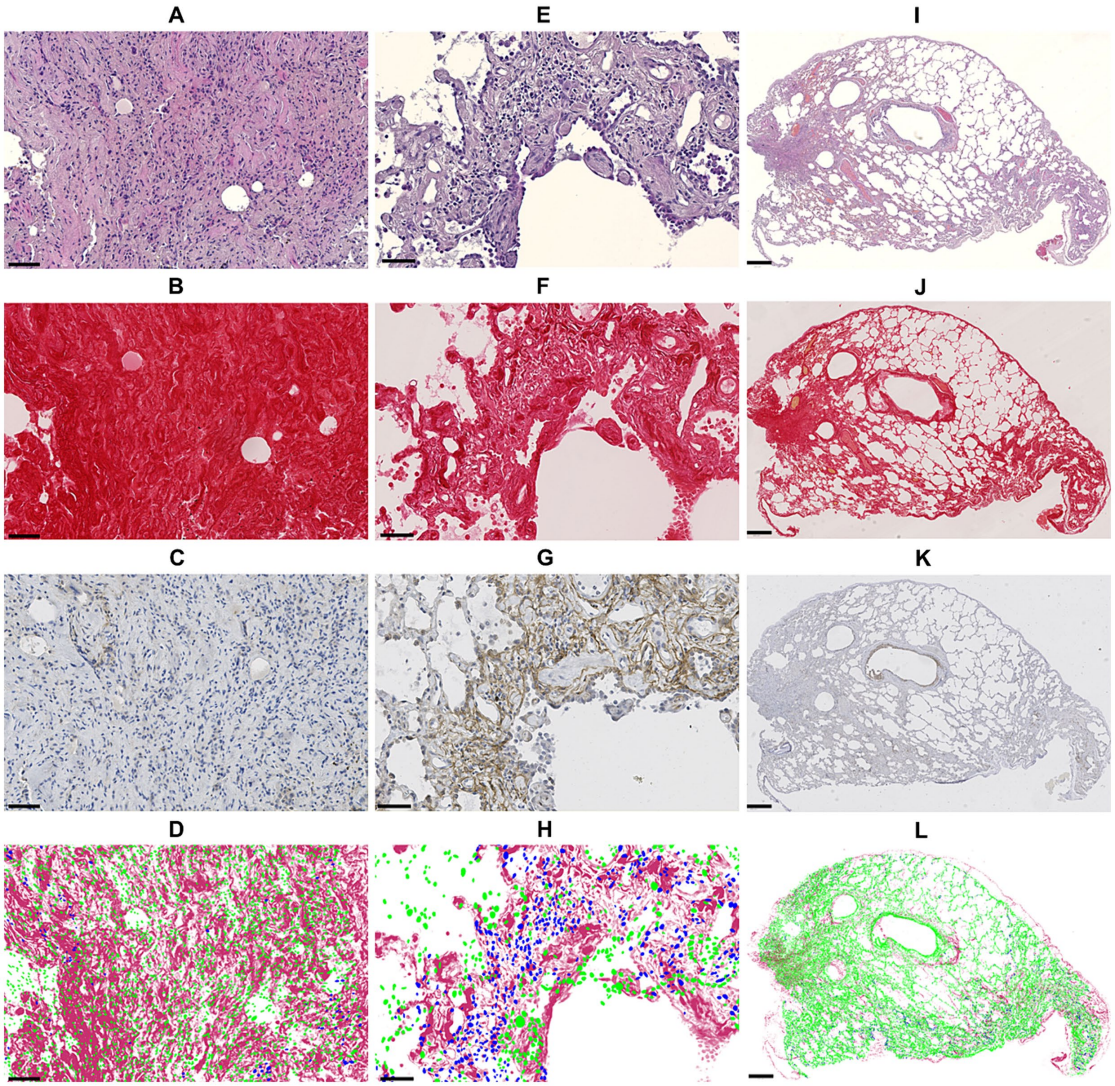
Panel showing sequential sections in HE staining (1st row), Picro Sirus red staining (2nd row), anti-FAP immunohistochemistry staining (3rd row) and the superimposition of cell detections (blue: FAP-positive, green: FAP-negative) based on anti-FAP immunohistochemistry onto Picro Sirus red-stained collagen (4th row). Images **(A–D)** show an area of strongly collagenic mature fibrosis with rare FAP-positive cells (bar: 50 μm). Images **(E–H)** illustrate an area of active fibrosis with low collagen content and numerous FAP-positive cells (bar: 50 μm). Images **(I–L)** show an entire section with mixed fibrosis pattern: few FAP-positive cells within highly collagenic mature fibrosis areas, from which less collagenic, FAP-rich areas extend (bar: 500 μm). FAP, fibroblast activation protein.

Using quantitative digital analysis, we analyzed 20 areas originating from 11 different cases which were previously attributed with various indices of FAP expression and of fibrosis activity. QuPath automated detection of collagen content in lesional areas accurately reflected the mature collagen fibers visualized by polarized light microscopy (Supplementary Figure 1). The mean collagen content was significantly higher in representative areas of mature fibrosis (32.95 ± 15.28%) than in representative areas of active fibrosis (11.20 ± 7.34%; *p* < 0.001; Figure 3A). This validated our visual, semiquantitative assessment of the maturity of fibrosis. The mean percentage of FAP-positive cells was significantly higher (*p* < 0.001) in representative areas of active (27.73 ± 8.57%) compared with mature fibrosis (9.64 ± 4.02%; Figure 3B). Visual superimposition of serial Picro Sirius red and FAP-stained sections (Figure 4) revealed that FAP-positive cells were rare within highly collagenic mature fibrosis areas (Figures 4A–D). However, FAP-positive cells were clustered in areas of active fibrosis within alveolar septa (Figures 4E–H) or dispersed at the periphery of mature fibrotic lesions, where collagen content is lower (Figures 4I–L).

### 3.3 Plasmatic FAP quantification

**Figure 5:**
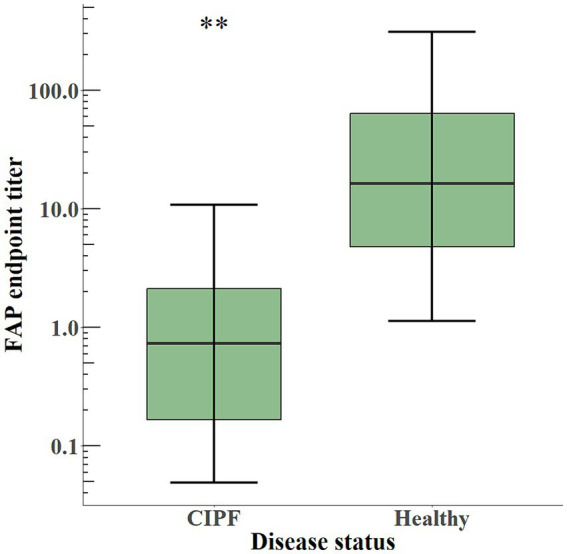
Box-and-whisker plot of plasma levels of soluble FAP in dogs with CIPF (*n* = 6) and in healthy dogs (*n* = 9). The box represents the median and interquartile range, and the whiskers represent the values within 1.5 times the interquartile range. Significance level: ** indicates a *p*-value below 0.01. FAP, fibroblast activation protein; CIPF, canine idiopathic pulmonary fibrosis.

The plasmatic levels of soluble FAP, as illustrated in Figure 5, were significantly lower (*p* < 0.01) in WHWTs with CIPF (EPT 0.74 [0.24–2.38]) than in healthy dogs (EPT 16.50 [4.78–63.84]).

## 4 Discussion

In this study, we established a semiquantitative scoring system destined to evaluate the severity and activity of fibrosis on histopathological sections of CIPF, which in this study exhibited mild to severe fibrosis. As expected, the FAP IHC study revealed that healthy lung sections were FAP-negative, and that cancer-associated fibroblasts from lung tumors were strongly positive. In the majority of CIPF samples, FAP was expressed by cells consistent with fibroblasts at various indices and only 2 samples were negative. FAP expression correlated weakly with fibrosis severity but highly with fibrosis activity. Indeed, automated image analysis detected a higher percentage of FAP-positive cells in areas of active fibrosis. It is also noticeable on superimposition of images that FAP-positive cells are located at the periphery of mature fibrosis lesions and clustered in areas of active fibrosis. Another finding of this study is that plasmatic FAP was significantly lower in WHWTs affected with CIPF compared with healthy dogs.

We established a system of scoring of the histological severity and activity of fibrosis in CIPF. This scoring system aimed to objectively distinguish lung biopsies of old healthy dogs from mild CIPF as well as mild from moderate and severe cases of CIPF. While attributing scores, a particular consideration was given to the distinction between active (immature) and inactive (mature) fibrosis for the purpose of subsequently establishing correlation with FAP IHC analyses. The Ashcroft scoring system (43) used for IPF cannot be applied to CIPF as human and canine IPF do not have the same histological pattern. Indeed, in humans, IPF is characterized by usual interstitial pneumonia (UIP) (39, 44). UIP diagnosis is based on patchy dense fibrosis accompanied by architectural distortion (with destructive scarring and/or honeycombing) with a predilection for subpleural and paraseptal parenchyma, and the presence of fibroblast foci (39, 44). CIPF shares features with UIP but also with non-specific interstitial pneumonia, which is characterized by diffuse interstitial fibrosis (37, 38). Indeed, in WHWTs affected with CIPF, histology reveals a mild to moderate diffuse mature interstitial fibrosis with multifocal subpleural or peribronchial areas of accentuation. Besides, no fibroblast foci are described (37, 38). The absence of fibroblast foci, as well as the high heterogeneity of fibrosis within a lung biopsy from a CIPF case, also prevented the application of scoring systems used for IHC studies of human IPF sections. This underlines the need for a specific scoring system adapted to dogs, as presented here.

This study confirmed the presence of FAP in lung biopsies from WHWTs affected with CIPF, and its absence in normal lungs, as anticipated from studies of human IPF (8, 9). Based on the assessment of their morphology and localization, FAP-positive cells appeared as fibroblasts. Nonetheless, the simultaneous expression of other fibroblast markers would be needed to confirm with certainty the precise identity of FAP-positive cells, although previous studies in human IPF reported a FAP expression restricted to activated fibroblasts (8, 9). The majority of cases were assigned a low FAP expression index according to the semiquantitative scoring system. This outcome, which might initially appear disappointing, is actually due to the fact that the scoring system assesses the overall expression across the entire section since CIPF exhibits a more diffuse interstitial fibrosis pattern than UIP (38). As a whole section of CIPF biopsy can be highly heterogeneous, with varying ratios of mature and active fibrosis, this heterogeneity significantly influences the proportion of the section occupied by FAP-positive areas.

In cases of CIPF, the FAP expression index exhibited a mild correlation with the severity score of fibrosis, aligning with findings from previous studies that have explored the association between FAP expression and fibrosis severity at both histological and clinical level in humans (8, 9). Nevertheless, a good correlation emerged when focused on the activity of fibrosis in lung biopsies. In UIP, the histologic pattern of human IPF, FAP expression is restricted to areas of ongoing tissue injury (8). FAP is indeed strongly expressed in fibroblast foci, which are interstitial clusters of proliferating fibroblasts and myofibroblasts near the injured alveolar epithelium (8). Despite the absence of fibroblast foci in CIPF, it is consistent that FAP is mostly expressed in highly fibroblastic active areas, and not in poorly cellular regions that are consolidated by dense amounts of collagen fibers. The identified positive correlation between FAP expression and fibrosis activity underscores FAP’s potential as a promising marker for fibroplasia, providing substantial support for the hypothesis that FAP plays a crucial role in the pathophysiology of the disease.

Automated quantitative image analysis technologies were used to confirm these results with a more sensitive and objective method. Digital image analysis using artificial intelligence solutions is emerging in the field of histopathology and IHC, providing promising techniques for scoring quantification of tissue fibrosis in human IPF or to quantify FAP positivity in IHC images (9, 43). Such quantifying tools allowed us to validate our semiquantitative scoring system of fibrosis used to select areas by their representativity of a type of maturity of fibrosis (active or mature) with a precise quantification of the collagen content of the area, which is an established marker of mature fibrosis (43). Then, it was confirmed that the proportion of FAP-positive cells was significantly higher in areas occupied by active fibrosis than in those occupied by mature fibrosis. Digital superimposition of FAP-positive cell detections onto Picro Sirius red stained sections allowed us to witness the spatial distribution of FAP-positive cells in relation to the lesions of fibrosis within the sections. FAP-positive cells, consistent with activated fibroblasts, are sparse within mature lesions and are mainly scattered in alveolar septa that are not yet burdened by large layers of collagen fibers. FAP-positive active fibrosis areas seem to be located in the periphery of mature lesions. In this view, FAP-positive cells appear to play a driving role in fibrosis.

The small cohort of primary pulmonary adenocarcinomas used in this study showed a strong expression of FAP by cancer-associated fibroblasts, and occasionally by cancer cells themselves, aligning with expectations based on both veterinary and human literature (17, 26, 27). This provides a foundation for potential extended investigations about the prognostic significance of FAP in lung cancer. Indeed canine lung cancers of advanced stage still carry a poor prognosis and could benefit from novel therapeutic strategies (45, 46).

This analysis revealed lower plasmatic FAP levels in WHWTs affected with CIPF compared with healthy dogs. To date, the variation of plasmatic FAP in humans affected with IPF is not known. However, it has been studied in various other conditions, notably in patients with cancer (18, 20, 24), inflammatory bowel disease (47) or acute heart failure (48), who also exhibited a lower plasmatic FAP concentration compared with healthy volunteers, despite an overexpression of FAP in diseased tissues. The reason for such decrease, as well as the source of the soluble form of FAP, are still unknown, including whether it results from shedding from the cellular membrane or from alternative splicing (7, 49, 50). Current hypotheses suggest that multiple organs may contribute to a low basal level of circulating FAP, which would decrease due to a systemic reaction to the disease (20, 48, 51). Interestingly, other studies showed an increase of circulating FAP in patients with liver fibrosis and support the hypothesis that the liver constitutes a major source of elevated circulating FAP (7, 52, 53). Further studies on a greater number of cases are thus required to explain why circulating FAP is decreased in CIPF.

This study of plasmatic FAP concentrations is based on a small number of dogs which could expose us to sampling biases. Furthermore, due to the nature of the selection criteria for blood donation (dogs of less than 10 years old and more than 20 kg), the control group is not matched for age and breed with the study population. Nonetheless, a strong association between age and circulating FAP levels has never been established in existing literature (32). However, it appears that various conditions can influence the level of plasmatic FAP, such as malignant, inflammatory, metabolic, cardiac, or other organs fibrotic conditions (24, 47, 48, 52, 53). Although plasmatic FAP appears significantly decreased in dogs with CIPF, we do not believe that it would constitute a useful biomarker of CIPF since it does not seem specific to the disease.

As perspectives, the sensitivity of FAP to identify active fibrosis specifically localized within the lungs in cases of CIPF can be harnessed by FAP-targeted PET examinations. FAPI-based PET/CT are emerging in preclinical and clinical studies on interstitial lung disease (such as IPF) or cancer and are presented as non-invasive tools to monitor disease progression or response to treatment (31, 54). This promising technique would allow to assess FAP expression in dogs *in vivo* and thus enable an early detection of CIPF and evaluation of progression or response to treatment. In this field, FAP-targeted therapies (such as anti-FAP CAR-T cells or FAPI-based theragnostic) emerge as promising prospects, given the current lack of available treatments for CIPF. Considering that CIPF is regarded as a spontaneous preclinical model of IPF, human patients could also gain advantages from these findings.

In conclusion, this study shows new insights into the pathology of CIPF by describing the cellular expression of FAP in progressing active immature lesions of fibrosis. These findings position tissular FAP – but not plasmatic FAP – as a promising marker of activity of the disease, which could be exploited by multiple diagnostic and therapeutic applications.

## Data availability statement

The raw data supporting the conclusions of this article will be made available by the authors, without undue reservation.

## Ethics statement

The animal studies were approved by the Animal Ethics Committee of the University of Liège. The studies were conducted in accordance with the local legislation and institutional requirements. Written informed consent was obtained from the owners for the participation of their animals in this study.

## Author contributions

ERi: Conceptualization, Data curation, Formal analysis, Investigation, Methodology, Validation, Visualization, Writing – original draft, Writing – review & editing, Resources. CM: Resources, Writing – review & editing, Formal analysis, Methodology, Software, Visualization. AF: Resources, Writing – review & editing. ERo: Resources, Writing – review & editing. PJ: Formal analysis, Investigation, Methodology, Writing – review & editing. EP: Conceptualization, Investigation, Methodology, Resources, Supervision, Writing – review & editing. M-MG: Conceptualization, Formal analysis, Investigation, Methodology, Resources, Supervision, Validation, Writing – review & editing. TM: Conceptualization, Methodology, Resources, Supervision, Writing – review & editing. CC: Conceptualization, Funding acquisition, Project administration, Resources, Supervision, Writing – review & editing, Validation.
