# Supplementary material for: Fibroblast activation protein is a cellular marker of fibrotic activity in canine idiopathic pulmonary fibrosis
Source: Front Vet Sci. 2024 Jul 31;11:1416124. doi: 10.3389/fvets.2024.1416124 (PMC11346374; doi:10.3389/fvets.2024.1416124)
Supplement: Supplementary file 1 [file Data_Sheet_1.PDF]

Supplementary Material

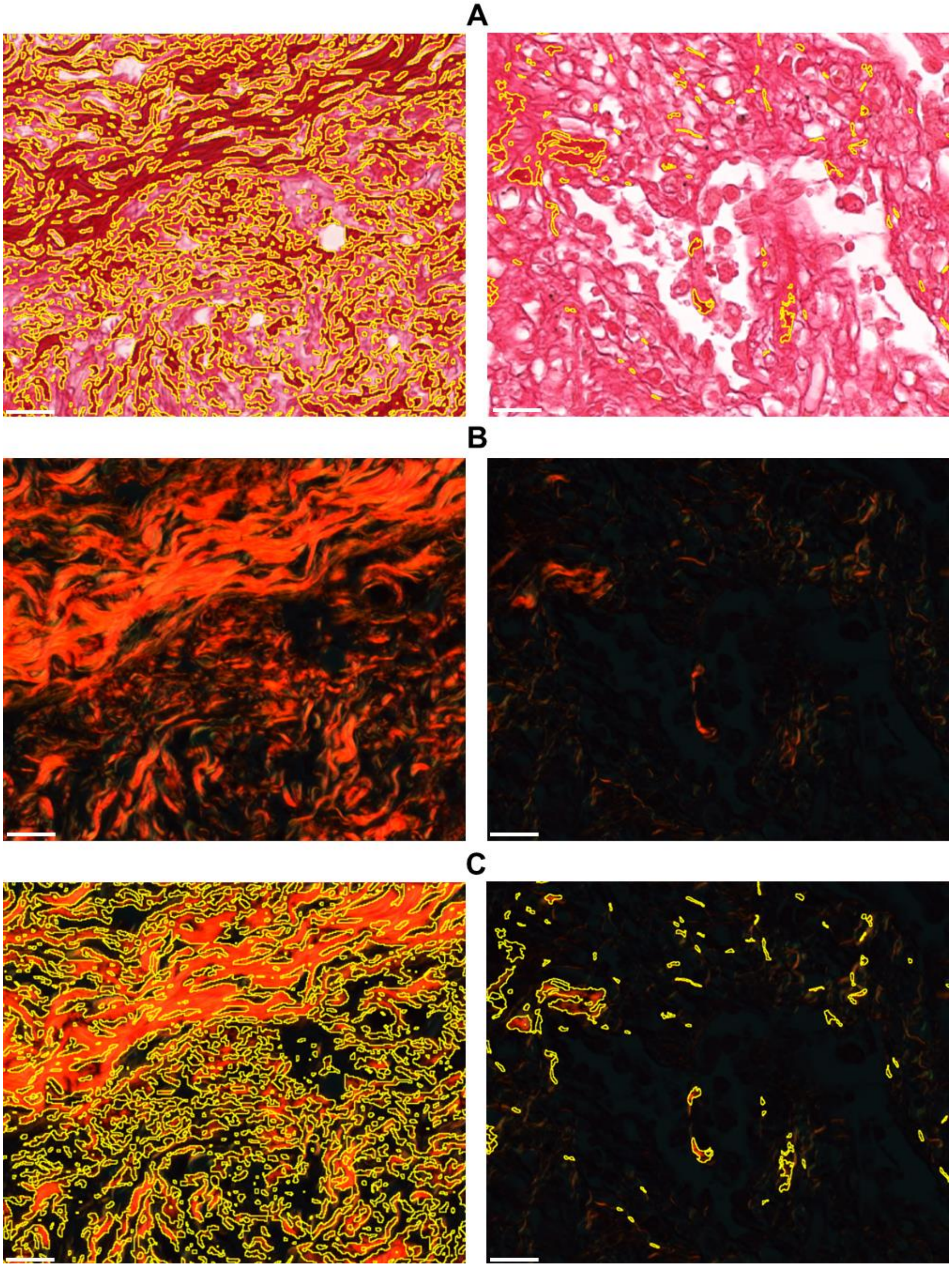

Supplementary Figure 1. Panel illustrating the accuracy of QuPath automated detection of collagen within areas of either mature fibrosis (left column) or active fibrosis (right column). (A) Collagen detections (outlined in yellow) in Picro Sirius red-stained sections digitalized in brightfield. (B) Visualization of collagen fibers within the same area digitalized under polarized light. (C) Superimposition of collagen detections onto polarized light images (bar: 20 µm).
